# Perceptions of Empathy Among Nursing Assistants in Long-Term Care Facilities: A Qualitative Study

**DOI:** 10.1155/2024/3072064

**Published:** 2024-11-08

**Authors:** Hsiao-Ling Hu, Chien-Lin Kuo, Shou-Yu Wang, Yeu-Hui Chuang

**Affiliations:** ^1^School of Nursing, College of Nursing, Taipei Medical University, Taipei, Taiwan; ^2^Tri-Service General Hospital Songshan Branch, Taipei, Taiwan; ^3^Department of Allied Health Education and Digital Learning, National Taipei University of Nursing and Health Sciences, Taipei, Taiwan; ^4^School of Nursing and Midwifery, Faculty of Health and Medicine, University of Newcastle, Callaghan, Australia; ^5^School of Health, Faculty of Medicine and Health, University of New England, Armidale, Australia; ^6^Department of Nursing, Wan Fang Hospital, Taipei Medical University, Taipei, Taiwan; ^7^Research Center in Nursing Clinical Practice, Wan Fang Hospital, Taipei Medical University, Taipei, Taiwan

**Keywords:** empathy, long-term care facility, nursing assistant, perception, qualitative study

## Abstract

**Background:** Empathy is a fundamental component of the therapeutic relationship between healthcare providers and patients and has the potential to yield significant benefits, including heightened care satisfaction, enhanced care quality, and improved mental well-being for patients. As nursing assistants (NAs) are primary direct care providers in long-term care facilities (LTCFs), it is necessary to understand NAs' views on empathy in their care provision for residents, but a gap exists in the literature regarding NAs' perspectives on empathy in this context.

**Aim:** In this study, we aimed to explore perceptions of empathy among NAs in LTCFs.

**Research Design:** A qualitative approach was applied in this study.

**Participants and Research Context:** Through purposive and snowball sampling, 18 NAs who provided care for residents in six LTCFs in Taiwan were recruited. Face-to-face in-depth interviews were conducted using a semistructured interview guide, and data were analyzed using a content analysis.

**Ethical Considerations:** Ethical approval was obtained from the Institutional Review Board. All participants signed a written informed consent form prior to the interview.

**Results:** Four themes encompassing 11 categories were generated including “being fundamental to caregiving to establish trust and enhance quality care,” “practicing altruistic behavior in the pursuit of ethical caregiving,” “cycling empathic flow in care,” and “facing challenges in delivering empathetic care.”

**Conclusions:** NAs in LTCFs viewed empathy as an essential element of the caregiving process. They saw empathy as selfless care, assistance to others, and alleviation of residents' suffering. Understanding residents' emotions, thoughts, and needs, active listening and compassionate engagement were vital to expressing empathy. However, NAs faced challenges in expressing empathy due to demanding tasks, time limitations, and insufficient knowledge and communication skills. In addition, emotional desensitization further complicated the provision of empathic care. The findings of this study can provide information for nurse managers and directors to understand NAs' perspectives on empathy and difficulties when providing empathic care in the long-term care context.

## 1. Background

Empathy is the comprehension of another person's experiences and feelings, allowing one to feel as if they were one's own, while still preserving the essential quality of “as if” [1]. However, empathy remains incomplete without subsequent responses or actions following understanding and recognition of someone's circumstances and emotions. It necessitates both the awareness and provision of responses. Barrett-Lennard [2] introduced the concept of an “empathy circle” consisting of three phases: inner resonation, where another person's expression resonates within oneself; delivering understanding to the person; and raising awareness of that understanding by that person. As a result, empathy entails the ability to comprehend an individual's circumstances, thoughts, and emotions; communicate and validate those components; and take actions based on that understanding [3].

Empathy is a cornerstone of the therapeutic relationship between healthcare providers and patients. Providing empathetic care by healthcare professionals is associated with various positive outcomes, including enhanced care quality [4], heightened patient satisfaction, increased patient enablement, reduced patient anxiety and distress [5–7], and promotion of patients' psychological well-being. Furthermore, such empathetic care contributes to the professional satisfaction of healthcare providers and mitigates the risk of job burnout [8, 9].

With aging populations on the rise, the number of older adults facing physical and cognitive function impairments is also increasing. Consequently, admission to long-term care facilities (LTCFs) has become a common option for older adults by their family members in Taiwan when caregiving is not readily available. In LTCFs, nursing assistants (NAs) are primary care providers. There were about 40,117 NAs in Taiwan's LTCFs in 2023, and they comprised 61.5% of the LTCF workforce [10]. Staffing ratios in Taiwan vary depending on the type of residential institution. For example, in nursing homes, the standard NA-to-resident ratio is 1 : 5 across all shifts [11]. In long-term care institutions, this ratio is maintained at 1 : 5 during daytime hours but changes to 1 : 15 for nighttime staff. In nursing institutions, the ratio is set to 1 : 8 for daytime shifts and increases to 1 : 25 for nighttime shifts [12]. However, since the implementation of “Taiwan's Long-term Care Plan 2.0,” many NAs have transitioned from residential institutions to home care due to better pay and more flexible schedules, leading to staffing shortages and recruitment challenges within these institutions. Sabbe et al. [13] mentioned that staff shortages in LTCFs increased NAs' responsibilities, leading to inadequate resident care. Rising work pressure also causes more absenteeism, further worsening the shortage and increasing workloads. This increasing burden may impact NAs' ability to provide empathic care that is essential to residents' well-being.

NAs shoulder the bulk of direct care responsibilities within LTCFs. A study revealed that NAs dedicate an average of 2.1 h per day to care for each resident [14]. This underscores the critical role of NAs, who maintain the most frequent and prolonged interactions with residents in these institutions. Consequently, NAs are required not only to possess accurate knowledge and caregiving skills but also to exhibit positive attitudes and attributes. Empathy is one of the essential attributes and plays a crucial role in establishing trust-based relationships with residents, fostering an environment where residents feel respected, cared for, and loved. Although the importance of empathy is widely recognized within the caregiving context, the majority of studies focused on issues around empathy in nursing students [15–18] or nurses in acute care settings [19–22]. How NAs in the long-term care context grasp empathy and put it into practice as well as their thoughts and feelings about it are still lacking. To bridge this gap, this study aimed to explore NAs' perceptions of empathy in LTCFs.

## 2. Methods

### 2.1. Research Design

In this study, we employed a qualitative approach to understand perceptions of NAs regarding the expression of empathy in LTCFs.

### 2.2. Research Setting and Participants

Purposive sampling and snowball sampling were applied. Inclusion criteria were participants who (1) had a NA license, (2) were at least 20 years old, (3) had worked in a LTCF for over 3 months, (4) could communicate in Chinese or Taiwanese, and (5) were willing to share their experiences. NAs who worked part-time were excluded. The sample size was determined based on the principle of data saturation [23], which occurs when no new information is encountered during data collection. In the current study, data saturation was reached after 18 participants in six LTCFs in Taiwan were recruited and interviewed.

### 2.3. Instruments

All interviews were conducted by the first author who had 5 years of research experience in qualitative studies and 4 years of nursing experience in long-term care. A face-to-face in-depth interview was performed using a semistructured interview guide. Prior to data collection, three NAs were interviewed to assess whether the participants understood the questions and if the questions were effective in achieving the research aim and to allow the researcher to practice interview skills. Interviews were conducted in a private space where participants felt comfortable, such as a discussion room or the participant's home. Interviews were recorded with a digital recorder, and recorded interviews were transcribed into a text file within 72 h after an interview. Notes were used to record participant's facial expressions and body language during the interview and the reflections and thoughts of the researcher. Each NA was interviewed once, and the interviews lasted for about 30–108 (mean = 52.3, SD = 21.5) minutes in this study. Information on demographic characteristics was obtained after the interview.

### 2.4. Data Collection

After obtaining approval from the Institutional Review Board (IRB), the researcher contacted the LTCFs by phone. After explaining the study purpose and methods, the head nurse or director of a LTCF introduced NAs who met the inclusion and exclusion criteria to the researcher. In addition, using snowball sampling, participants also introduced other NAs to join the study. The researcher first explained the study to all eligible NAs. If they wanted to participate in the study, they signed an informed consent form before the interview began. Data were collected from March to October 2022.

### 2.5. Data Analysis

A content analysis was used to analyze the data [24–27]. Steps of the data analysis included the following: (1) repeatedly reading the transcripts, (2) identifying meaning units and then condensing them into condensed meaning units, (3) labeling condensed meaning units using codes, (4) grouping codes with similar attributes into categories, and (5) grouping similar categories together to generate themes. Two researchers separately analyzed the data and then discussed the results until they reached a consensus.

### 2.6. Rigor of the Study

We used four criteria to enhance the rigor of this study [28]. To enhance the credibility of the study, member checking was performed with two participants who were provided the findings of the study. In addition, researchers had been trained in qualitative research for several years. As to transferability, the study setting, sampling, data collection methods, and data analysis were thoroughly described. Moreover, researchers maintained an audit trail by giving a vivid description of the research process from the inception of the study to the reporting of the study findings to enhance its dependability. As to confirmability, two researchers analyzed the data, and an audit trail was carefully documented.

### 2.7. Ethical Considerations

Ethical approval was obtained from the Taipei Medical University-Joint Institutional Review Board (N202203004). Participation in this study was entirely voluntary. All participants were clearly informed that they could cease their involvement at any stage of the study and face no adverse effects. In addition, all participants signed a written informed consent form prior to the interview.

## 3. Results

### 3.1. Demographics and Characteristics of Participants

The average age of the 18 NAs was 50.3 ± 12.8 years. The majority of participants were female (77.8%), had a high school diploma (72.2%), were married (72.2%), and had no religious beliefs (72.2%). On average, they had worked in LTCFs for 9.7 ± 5.8 years (Table 1).

### 3.2. Perceptions of Empathy Among NAs

Four themes generated in this study included “being fundamental to caregiving to establish trust and enhance quality care,” “practicing altruistic behavior in the pursuit of ethical caregiving,” “cycling empathic flow in care,” and “facing challenges in delivering empathetic care” encompassing 11 categories (Table 2).

#### 3.2.1. Being Fundamental to Caregiving to Establish Trust and Enhance Quality Care

Empathy is an important attribute in healthcare. NAs recognized that empathy is essential and plays a crucial role when providing care for residents, because through empathy, a trusting relationship with residents can be established, stronger connections with residents can be formed, and a reciprocal cycle of care can be achieved. Empathy naturally facilitates these outcomes by enabling NAs to connect with residents on a deeper level, which leads to enhanced trust and a higher quality of care. This theme consisted of three categories as follows: “fulfilling responsibilities,” “building trusting relationships,” and “creating a positive reciprocal cycle of care.”

##### 3.2.1.1. Fulfilling Responsibilities

NAs expressed that providing care with empathy is a fundamental aspect of NAs' roles and responsibilities that is deeply embedded in the caregiving process.

I'm doing my best to care for you (a resident) and make sure you (a resident) are well. … As long as you work for one day, you must do your job well. (*C, female, 61 years old*)

Empathy is about helping older individuals who can't take care of themselves. We should do our best to give them the best assistance in their daily lives and care. (*H, female, 23 years old*)

##### 3.2.1.2. Building Trusting Relationships

Recognition of empathy by NAs as a method to build trust with residents is crucial for fostering a caring and supportive environment in LTCFs. The following quotes reflect how empathy can be the foundation for building trusting relationships, leading to more effective and harmonious care.

When interacting with him (a resident), slowly, I feel like... as if when I do things (in accordance with his habits), they will trust me more, and the caregiving process becomes smoother. (*B, female, 43 years old*)

He (a resident) knows that I understand what he's thinking, so we're like buddies... He feels a sense of trust towards me because I support him, I understand him, and I know what his next move and needs are. (*F, female, 54 years old*)

##### 3.2.1.3. Creating a Positive Reciprocal Cycle of Care

By incorporating empathy into their practice, NAs can establish meaningful connections and improve resident compliance with treatment plans and cooperation during daily care routines. Residents are more likely to follow instructions and actively participate in their own care when they trust their caregivers, resulting in a positive reciprocal cycle of care.

In the beginning, I first responded to his condition and his needs, and then, as we built a trusting relationship, I gradually adjusted to his preferences. It's like once trust was established, he became more receptive to the way I do things, meaning he could accept whatever approach I took. (*F, female, 54 years old*)

By paying attention to those details that make patients (residents) feel we are sincere, they actually give us very positive feedback. That kind of two-way communication feels very benevolent and comforting. Interactions become smooth and harmonious. (*O, female, 63 years old*)

These quotes illustrate how empathy fosters a positive reciprocal cycle of care, where trust and cooperation enhance the caregiving experience for both residents and caregivers.

#### 3.2.2. Practicing Altruistic Behavior in the Pursuit of Ethical Caregiving

Some NAs saw empathy as an instinctive human trait. When NAs observed older residents experiencing dependency, suffering, helplessness, and negative emotions, they felt a genuine desire to help and improve their situation, resulting in altruistic actions. These selfless behaviors reflect the ethical principles of caregiving, where the well-being of residents is prioritized through compassionate and morally driven care. This theme consisted of two categories as follows: “exhibiting compassion” and “practicing benevolence.”

##### 3.2.2.1. Exhibiting Compassion

“Compassion is inherent in all human beings” according to Mencius [29] and can describe the perceptions of NAs. They expressed that empathy is inherent in humans, and everyone possesses a sense of empathy. When NAs observed residents' difficulties, they had the desire to take action to alleviate the suffering and hardship of residents.

I would feel sad if I were in the same situation... I feel that they are working very hard, lying there is truly tough for them, so I will treat them well. (*M, female, 52 years old*)

In fact, helping residents alleviate suffering seems like an instinct. When you have someone suffering and in need of care around you, naturally you will care for them and feel the urge to lend a hand, take care of them… (*O, female, 63 years old*)

##### 3.2.2.2. Practicing Benevolence

In addition to compassion, some NAs expressed that empathic care is a kind of benevolent practice. They also viewed taking care of older residents as an act of kindness. They considered caregiving as a way to contribute to society and help others and derived a sense of spiritual fulfillment from it.

When he (a resident) is in need, I can help lighten his burden. Besides helping him, I also gain some energy myself... (*M, female, 52 years old*)

Treating the elderly with kindness and respect at work is truly an embodiment of compassion. Assisting them while we still possess the ability is a means of contributing to society, and personally, it's a method of ensuring that the spirit of love and care endures. (*O, female, 63 years old*)

#### 3.2.3. Cycling Empathic Flow to Provide Care

NAs addressed the cycle of empathy in the caregiving process. With empathic flow, NAs could acquire the ability to correctly understand and recognize residents' feelings, make an effort to understand their situation and suffering, and appropriately respond to them with compassion and understanding. During caregiving, the steps of empathic flow continually reinforce and build upon each other, creating a dynamic process that deepens the connection between NAs and residents. This theme consisted of three categories as follows: “understanding residents' feelings,” “putting oneself in someone else's shoes,” and “having an empathetic approach.”

##### 3.2.3.1. Understanding Residents' Feelings

NAs emphasized the importance of understanding residents' feelings as a crucial aspect of expressing empathy. This involves a cognitive aspect of empathy, where NAs are attentive and perceptive to the emotions and experiences through which residents are going.

Because I have been a patient before, I understand how uncomfortable residents can feel. (*G, female, 48 years old*)

In particular, his family members seem to very rarely come... He (a resident) hopes for companionship from others, and every time I see a sense of loneliness in his eyes, it is because of other visiting families that are not his own. (*Q, male, 25 years old*)

##### 3.2.3.2. Putting Oneself in Someone Else's Shoes

This goes beyond understanding on a cognitive level and involves the emotional aspect of empathy. NAs try to imagine what it feels like to be in a resident's situation, and emotionally connect with their experiences. It is an active effort to see things from residents' perspectives and feel the emotions they are experiencing.

People often say to treat patients like family, but I think we should go beyond that... We should treat patients as if they were ourselves... If you were in their position, how would you want to be cared for? Treat them the same way. (*P, male, 64 years old*)

Put yourself in their shoes. If you were in their position, how would you want others to care for you? Treat the grandparents (residents) the same way. (*I, female, 46 years old*)

##### 3.2.3.3. Having an Empathetic Approach

NAs suggested that the ways of responding to residents' feelings and situations with compassion were effective communication, being there, and being patient.

###### 3.2.3.3.1. Effective Communication

NAs stressed that effective communication with residents, utilizing language, facial expressions, and nonverbal cues to convey their understanding and respond to residents is essential during the caregiving process.

When older residents can speak, we communicate with them using words. It is essential to take the time to observe what we have noticed and communicate with them to confirm if it is what they want. (*A, female, 37 years old*)

The resident with dementia I cared for was unable to articulate his thoughts using language. Therefore, I understood his needs by observing his body language. When I sensed that he was uncomfortable, I responded to him in my own way. If he felt dizzy or unwell, then I provided massage oil to help alleviate his discomfort. Through this way of communication, he could feel that I understood his needs, even though he couldn't express himself or take action on his own. (*F, female, 54 years old*)

###### 3.2.3.3.2. Being There

NAs need to provide attentive companionship in order to make residents truly feel the caregivers' presence. Attentive companionship is a precious act of empathy.

Listen and pay attention to residents and closely observe to understand their needs and feelings. (*J, female, 59 years old*)

Empathy is crucial in caregiving, as it requires attentive companionship, active listening, and meaningful interactions. (*A, female, 37 years old*)

###### 3.2.3.3.3. Being Patient

NAs mentioned that being patient is also very important to expressing empathy. The caregiving process requires time and energy, so NAs need to have enough patience, especially when it comes to assisting residents with daily activities or addressing their needs and emotional concerns, which may take a lot of time.

We might have to be patient and give him (a resident) a bit more time to respond... (*M, female, 52 years old*)

We need to find ways to overcome it by reducing and prioritizing other tasks and dedicating time specifically for him. It's essential to plan and allocate the necessary time for his (resident's) needs throughout the process... (*R, male, 72 years old*)

#### 3.2.4. Facing Challenges in Delivering Empathetic Care

In interactions with residents, it might be difficult for NAs to adequately express empathy, due to time constraints, the workplace atmosphere, lacking relevant empathetic knowledge and communication skills, and overlooking residents' feelings. These challenges hinder the delivery of empathetic care, making it harder for NAs to effectively meet residents' emotional needs. This theme consisted of three categories as follows: “having a willing heart, but being unable to express empathy,” “lacking empathy-related knowledge and communication skills,” and “becoming desensitized and subsequently overlooking this aspect.”

##### 3.2.4.1. Having a Willing Heart, but Being Unable to Express Empathy

Heavy workloads and time constraints make it challenging for NAs to express empathy. Despite recognizing the importance of empathy, they often rush through tasks and hesitate to spend extra time with residents due to time pressures.

In fact, we have a heavy workload, and each of us has to take care of a significant number of residents. If the beds are full, one person may have to attend to as many as 10 residents, which means that the time allocated to each person is limited. (*G, female, 48 years old*)

We prioritize residents' needs when we have time, but it becomes challenging when we're busy. Sometimes, we can't respond promptly to their call button and have to prioritize other tasks. (*B, female, 43 years old*)

##### 3.2.4.2. Lacking Empathy-Related Knowledge and Communication Skills

NAs expressed their concerns about their limited understanding of empathy and lack of communication skills. These limitations make it challenging for them to effectively demonstrate empathy, as they struggle to fully comprehend residents' feelings and appropriately respond to them.

When the older lady (a resident) gets confused, we struggle to communicate with her as she doesn't understand… Unfortunately, ignoring her becomes our only option… (*B, female, 43 years old*)

After spending a long time in this job, you will probably hear about some of the negative emotions and experiences that the residents have. I feel that sometimes we really don't know how to respond to them... When we don't know how to respond, we might end up ignoring them. (*M, female, 52 years old*)

##### 3.2.4.3. Becoming Desensitized and Subsequently Neglectful of This Aspect

Some NAs expressed that they had become desensitized toward residents who constantly repeated their stories or exhibited repetitive behaviors. As a result, they might neglect residents' needs or requests.

Empathy is strong at first, but it tends to wane over time. At first, you're patient, but over time, you begin to feel tired and helpless, especially if residents don't cooperate. (*B, female, 43 years old*)

Some residents frequently cry and express their loneliness due to the lack of family companionship. They have gotten used to saying those words, and we have also gotten used to hearing them, so we just ignore them. (*L, female, 42 years old*)

## 4. Discussion

In the current study, NAs perceived that empathy was fundamental to the nature of care and making altruistic actions during caregiving. They also identified the process of delivering empathic care and the challenges they faced as NAs in effectively providing empathic care.

As it is fundamental to caregiving to establish trust and enhance quality care, the natural and essential role of empathy is highlighted in caregiving. Empathy is a fundamental element of NAs' caregiving responsibilities, and it is crucial for building trust and delivering high-quality care. Through empathy, NAs establish positive interactions with residents, thereby cultivating a trusting relationship with them. This finding echoed the research conducted by Liu and Chang [30], which underscored the significance of empathy in the process of developing caregiving relationships between NAs and older residents. NAs gradually earn the trust of older residents, fostering a sense of security and forming robust interactive relationships. Similarly, Tan et al. [31] addressed how empathy was perceived as establishing a sense of trust and connection within patient–healthcare professional relationships, notably prominent in nurse-patient and nursing student–patient interactions. Two other studies indicated that empathy is a skill acquired through socialization that assists in the formation of connections during patient–healthcare professional interactions [32, 33]. Furthermore, the current study also revealed that some residents felt and accepted empathy from NAs, so they would like to empathize with NAs' situations. Consequently, NAs' caregiving process may become smoother and more harmonious, fostering a bidirectional empathetic feedback loop that attains a state of positive cyclic reinforcement, ultimately culminating in a realm of mutual trust and reciprocity.

NAs conceptualized empathy as a manifestation of altruism. For them, delivering empathic care means putting altruism into action. Observing the distress or suffering of another prompts a natural sense of compassion and a yearning to mitigate their difficult situation, echoing the sentiment proposed by Mencius that “compassion is inherent in all human beings” [29]. Baston et al. [34] also concluded that empathy leads to altruism. Altruism is marked by selfless actions directed toward others that originate from genuine goodwill and lead to bestowing benefits without expectations of reciprocation. This kind of motivation is the essence of altruism in order to enhance others' well-being [35]. Altruistic behaviors can help others gain happiness without expecting reciprocity or rewards from them in return [36]. NAs recognized that while engaged in the caregiving process, thoughts of how to assist residents in reducing their suffering naturally emerged upon witnessing residents' difficult situations. This constitutes the practice of empathy and exemplifies the embodiment of altruism. Scholars have found that altruism is a core nursing value, emphasizing its relevance to the quality of care received by patients [37, 38].

Cycling empathic flow in care includes understanding residents' feelings (emotional perspective), putting oneself in someone else's shoes (cognitive perspective), and employing an empathetic approach (behavioral perspective). NAs highlighted that the process of empathic flow includes drawing from their own personal experiences of illness or caregiving for loved ones to better grasp the residents' current situations and stepping into the residents' shoes to fully comprehend their emotions. NAs could subsequently translate their understanding and emotions into verbal, nonverbal, and actionable expressions to provide an empathic approach. NAs also emphasized the “empathetic approach” wherein acts of attentive companionship and listening become critical in expressing empathy. The act of attentively listening to residents' voices enables NAs to accurately empathize with their verbal and emotional expressions, thereby fostering effective communication. Similarly, Skovholt [35] proposed the concept of the “cycle of caring,” which examines the application of empathic presence in psychotherapy. Skovholt's model emphasized that interactions between psychotherapists and clients are a dynamic cyclical process. Psychotherapists engage in a continual process of understanding a client's situation, investing their own emotions and feelings, and ultimately achieving emotional separation to maintain their own mental and emotional well-being. In this study, we found that NAs experienced similar stages of empathic attachment and active involvement. However, emotional separation was not easy for them, because NAs provided continuous care for residents in LTCFs and engaged with them on a daily basis. As a result, achieving emotional separation was challenging due to the ongoing nature of their caregiving role. Some NAs did acknowledge that excessive emotional involvement could impact their own emotions and may even lead to intentions to leave the job. This needs to be further researched to understand the situation.

Khan et al. [36] proposed that empathy among healthcare professionals is a crucial factor in understanding patients' feelings and emotional states, thereby motivating healthcare providers to offer care and assistance. When healthcare professionals gain a deeper understanding of patients' suffering, they are more driven to help alleviate their pain. Other views by Douglas et al. [39] emphasized the critical importance of effective communication in aged care, highlighting the need for NAs to receive sufficient training, especially in cultivating communication skills. Developing verbal communication skills can improve NAs' interpersonal relationships with residents. In addition to effective communication skills, accompanying and active listening are also common approaches to providing empathetic care. Furthermore, being patient is an important attribute in practicing empathy. NAs emphasized the need to recognize their own emotions, remain objective, and exhibit patience throughout the caregiving process. Similar findings were echoed in a study conducted by Hong and Yao [40], where female home care providers perceived companionship to be one of the best forms of care provision. Tan et al. [31] also observed that communication skills constitute a crucial element in conveying empathy. Effective communication skills can be enhanced through training, but the attitudes of healthcare professionals must be genuine. Apart from verbal communication, nonverbal behaviors, such as simple gestures signifying genuine concern and care, play a significant role in conveying empathy. A study highlighted the importance of caregivers patiently communicating with residents to make them feel understood and secure during care provision [13].

Some reasons that NAs have difficulty engaging in empathic care include heavy workloads, limited time, a lack of sufficient knowledge of delivering empathy, and getting used to the suffering or situations of residents. A recent study showed that due to understaffing, NAs have heavy workloads and caregiving burdens [13]. While desiring to provide holistic care, time constraints often limit NAs to fulfilling only basic physiological needs and ignoring other care aspects. Abrahamson et al. [41] found that challenges faced by NAs include utilizing nonverbal communication, balancing personal care while maintaining professional boundaries, and addressing a clear lack of trust. Another study revealed that healthcare providers acknowledged the importance of empathy yet encountered challenges in its daily application due not only to inadequate time but also to insufficient empathy-related training [42]. This echoes the findings of the current study. Empathy training or cultivation is mainly emphasized for nursing students, nurses, or other healthcare professionals. In Taiwan, NAs have a different educational background and only receive 60 h of course training and a 30 h practicum before taking the NA certification exam [43]. Even worse, training programs for NAs primarily emphasize physical care. It is suggested that empathy cultivation by NAs should be included in their training programs and in-service education in the near future. In addition, healthcare professionals might experience “numbing” due to residents' repeated behaviors or requests, resulting in neglect or unresponsiveness to residents' needs. To prevent or decrease the desensitization or numbing of emotions of NAs, nurses and nursing managers should develop appropriate strategies to improve this situation [44].

### 4.1. Limitations

More than two-thirds of the participants were female (77%) in this study. However, the statistics reflect the reality of the gender distribution among NAs in LTCFs in Taiwan, because the majority of NAs in LTCFs are female, accounting for 84.3% of the workforce [11].

## 5. Conclusions

This qualitative study aimed to understand NAs' perceptions of the practice of empathy in providing care for older residents in LTCFs. The findings revealed that NAs consider empathy to be fundamental during caregiving and when practicing altruistic behaviors. They deliver empathic care through understanding residents' feelings, putting themselves in the residents' situation, and providing feedback. They need to have the ability to feel the situation experienced by residents and have empathic behaviors toward residents, resulting in the formation of empathic flow. Older residents can feel NAs' empathy through effective communication, being there with residents, and being patient. However, challenges such as limited time, lack of empathy-related knowledge and skills, and emotional desensitization due to getting used to residents' repeated behaviors or requests can hinder their practice of empathy. It is recommended that managers or directors in LTCFs acknowledge the challenges faced by NAs in practicing empathy, provide empathy training, and create a supportive environment to foster a culture of delivering empathic care, ultimately enhancing the quality of care provided to residents of LTCFs.

## Figures and Tables

**Table 1:** Characteristics of participants (*N* = 18).

Participant	Gender	Age (years)	Educational level	Marital status	Religious beliefs	LOW (years)
A	Female	37	High school	Married	Buddhism	8
B	Female	43	High school	Divorced	None	15
C	Female	61	Junior high school	Married	Taoism	19
D	Female	63	Junior high school	Divorced	Taoism	15
E	Female	52	High school	Married	None	15
F	Female	54	College	Married	None	2
G	Female	48	High school	Married	None	11
H	Female	23	High school	Single	None	2
I	Female	46	High school	Married	None	5
J	Female	59	High school	Married	None	14
K	Female	45	Junior high school	Married	None	7
L	Female	42	Junior high school	Married	None	9
M	Female	52	High school	Divorced	Buddhism	3
N	Male	56	High school	Single	Taoism	6
O	Female	63	High school	Married	None	18
P	Male	64	High school	Married	None	11
Q	Male	25	High school	Married	None	0.5
R	Male	72	High school	Married	None	14

*Note:* LOW, length of work experience in long-term care facilities.

**Table 2:** Themes and categories of perceptions of practicing empathy among nursing assistants.

Theme	Category
Being fundamental to caregiving to establish trust and enhance quality care	Fulfilling responsibilities
	Building trusting relationships
	Creating a positive reciprocal cycle of care

Practicing altruistic behavior in the pursuit of ethical caregiving	Exhibiting compassion
	Practicing benevolence

Cycling empathic flow in care	Understanding residents' feelings
	Putting oneself in someone else's shoes
	Having an empathetic approach

Facing challenges in delivering empathetic care	Having a Willing Heart, but Being Unable to Express Empathy
	Lacking empathy-related knowledge and communication skills
	Becoming desensitized and subsequently neglectful of this aspect

## Data Availability

Due to the sensitive nature of the data, access to the data is restricted.
